# Displacement in Place and the Financial Crisis in Lebanon

**DOI:** 10.1093/jrs/fead076

**Published:** 2023-10-24

**Authors:** Ali Ali

**Affiliations:** Geography Department, University of Sussex, Brighton BN1 9RH, UK

**Keywords:** Lebanon, financial crisis, displacement, immobility, forced migration, refugees

## Abstract

Displacement is underway in Lebanon after financial collapse, but not as events of migration, rather, as processual disruption to people’s lives that begins in place, preceding the potential outcome of forced migration. Financial collapse has shifted the population into extremes of constraint, dispossessing them of assets needed to live in valued ways. Widely circulated claims of an exodus are premature. Historic mass emigration from Lebanon occurred in times of capital availability whilst today’s financial collapse denies most people of the capital needed to emigrate. Migration remains limited to the few with social and cultural capital unaffected by the crisis. This article was prompted by the author’s observations of financial collapse whilst living in Lebanon in 2020 and long-standing engagement with the country. Regardless of whether mass emigration occurs, perhaps after the economy’s recapitalization, the displacement process already underway warrants attention from refugee and forced migration studies.

## Introduction

Lebanon is in turmoil. The state is on the brink of collapse (ICG 2021) and those who had assets have been robbed of them by a major swindle, a national Ponzi-scheme run by the central and private banks (Chaaban 2016; Halabi and Boswall 2019). People have been robbed of their savings, basic public services, and perhaps their futures. Despite Lebanon’s long history of emigration (Hourani and Shehadi 1992), most have been robbed of their capacity to address macro-economic collapse with the option, or capability, of emigration as a support or survival strategy. Emigration is an option for a small minority with economic, social, and cultural capital unaffected by the crisis. Media outlets amplify warnings of dramatic mass emigration with stories largely focusing on high-skilled professionals and entrepreneurs, or tragedies at sea of those who fail (AFP News Agency 2021; Chehayeb 2021; Collard 2021; Damaj 2021; Observer 2022). However, focusing on the small minority able to leave safely, or who have died trying to, renders less visible the major transformations underway in Lebanon displacing the population in place.

The crisis is humanitarian as well as economic, but could easily be overlooked in refugee and forced migration studies (RFMS) because, despite reports of an exodus, relatively few have left Lebanon. It is important to understand the impact of the crisis on Lebanon’s population regardless of present and future migration figures. A key argument of the article is that even if emigration increases, the analysis in the field should not focus only on those who leave, the conventional event-centred approach which speaks of displacement and forced migration interchangeably and therefore imprecisely. Rather, our attention should also focus on the acute transformations in Lebanon affecting those who remain, and which constitute displacement in place: that is, coercive processes that disrupt valued ways of living and functioning (Ali 2020, 2023) which begin before people leave their homes (Celestina 2016; Vaz-Jones 2018). The clock of displacement starts ticking, so to speak, before people leave (Celestina 2016), meaning before the 2019 collapse began, when warnings of looming financial crises were sounded (Alami 2018, Baumann 2019), if not earlier. Furthermore, the article shows that it is not only the macro-economic contraction’s severity causing displacement in place—GDP in 2021 being 42 per cent of 2018 figures (World Bank 2023)—but also the specific features of the financial collapse, and corrupt elites’ refusal to enact reforms. They severely limit the possibilities to emigrate. Despite media attention on departures and tragedies, it remains extremely difficult to leave.

## Methodology

This article was prompted by observations of the financial collapse’s impact on daily life. I lived in Beirut from early March to late June 2020, during Lebanon’s CV19 lockdown, and the currency’s freefall. It was not my first or last visit: I spent three months there in 2016 and made multiple short personal and research-related trips to Lebanon between 2000 and 2022. I arrived in March 2020 to conduct a different research project that was aborted after complying with my sponsoring university’s instructions to avoid face-to-face research at the pandemic’s onset. This research is not ethnographic, nor was that something not possible or desirable at the start of the pandemic. However, during daily routines in Lebanon, buying food, medication, buying lira, moving around the city and later the country, I heard people express their concerns. The pessimistic expectations of 2020 have been outpaced by the rapid decline in the three years that followed.

I lived in Geaitawi, a Christian neighbourhood in east Beirut, and listened to worries of shopkeepers and traders, students, staff at cafes and food outlets, about the currency’s freefall and their businesses, livelihoods, and education. Geaitawi has a mix of middle and working-class residents, lacking the wealth of nearby Achrafieh. While fluent in Syrian Arabic, but having Iraqi heritage, I spoke Iraqi Arabic to avert the hostility that many in Lebanon direct towards Syrians, something I have experienced speaking Syrian Arabic in Lebanon. Some Christians preferred to speak English when they learned I was from the UK. But I also spent time in Hamra, a mixed neighbourhood, and spoke at length with a Lebanese mobile phone vendor in Zahira between long walks across locked-down Beirut. I wondered if many Lebanese citizens would be emigrating again. Lebanon’s diaspora is huge, spanning continents for reasons including war, labour migration, entrepreneurship, and their combination (Hourani and Shehadi 1992). Yet I also learned from conversations and news reports that not only had lira savings lost their value, but that banks were denying people access to their dollar savings. Capital is a necessary resource for emigration. I was also learning about the knock-on effects of the financial collapse on their abilities to live in valued ways: their concerns about current and future affordability and access to food, education, and healthcare. Having to live this way was no accident. Their assets and future resources were being taken away by a more powerful agent. Banque du Liban, Lebanon’s central bank (BDL), has forced people to pay for a collapse it engineered and then exacerbated through deliberate inaction. People were living in intensifying constraint, their choices and their capacity for action were being restricted in ways evidencing structural coercion. This was indicative of the displacement process, something I had researched in contexts of armed conflict, aspects of which I have conceptualized at greater length elsewhere (Ali 2023). It became increasingly clear that the population was experiencing the pressures of displacement, but those who might wish to leave, or to send a member of their household abroad, had their capabilities severely diminished not only by economic contraction, but by intentional government inertia, including its refusal to implement capital control laws which were partly to blame for banks denying people access to their savings until a time when they had become worthless.

While discussion and analysis are informed by long-standing engagement with the country, including some consideration of the effects on its already precariously situated refugee populations, the article is primarily a ‘big-picture’ discussion of the collapse and how it has displaced the population of Lebanon. This is in part because of pandemic limitations on face-to-face research, but also because the situation has deteriorated rapidly from the major challenges I observed in 2020. My phone-credit vendor in Zahira’s concern at the time the lira sunk to 3000LL from 1500LL to the dollar, was that after its 50 per cent fall in value, he was effectively starting from scratch regarding his bank loan, half of which he had paid back after years of work. By the end of my four months in Lebanon in 2020, the lira fell shockingly to 10,000LL, 15 per cent of its 2019 value. Since February 2023, the lira has hovered around 90,000LL to the dollar, under 2 per cent of its pre-collapse value. My phone vendor was robbed not only of his assets, but of a future free of debt, and of the choice to invest income into something other than debt repayments.

## Outline of the Article

The article is structured as follows. First, I provide a succinct context to the financial collapse to explain its origins and magnitude beyond an acute economic contraction. I then explain why the widely circulated warnings of an exodus are imprecise, because migration today is limited to the few with forms of capital unaffected by the collapse. I show how the historical migrations to which they refer differ, particularly in relation to capital availability. I then set out what I mean by displacement as a process that begins in place, and how the financial collapse has led to the disruption of baselines relating to income, education, and healthcare, that are important to the current and future circumstances of Lebanon’s population. The article then provides examples of efforts to resist and cope with these displacement pressures, including political resistance and community and refugee initiatives, some of which I observed when visiting Lebanon in 2022, before drawing conclusions about displacement in place.

## Systemic Financial Collapse

Lebanon’s economic predicament has significance beyond a severe macro-economic recession. It is a systemic collapse exacerbated by the policies of BDL (Banque du Liban, Lebanon’s central bank). The collapse resulted from the failure of a Ponzi-like scheme between BDL and the largest private banks (Halabi and Boswall 2019). Political elites have significant ownership in 18 of 20 of Lebanon’s major banks (Chaaban 2016). They shaped a system through which major banks profited from lending to BDL at unmanageably high interest rates. The BDL continued to borrow money from them and foreign lenders, creating debts of 150 per cent of GDP in 2019. Private banks continued lending to BDL, receiving high interest rates. This system operated for years because billions in foreign currency flowed into Lebanon from those in the diaspora drawn to high returns on their savings, from remittances of Lebanese diaspora, from the external bailouts sent by oil economies including Saudi Arabia, Qatar, Kuwait and the UAE, and their high-spending tourists upon whom many businesses depended. A ‘perfect financial storm’ gathered (Alami 2018). A combination of political incompetence, unfavourable geo-political and regional economic conditions, sharply curtailed these sources of foreign currency replenishment. The government subsequently defaulted on a $1.2 bn Eurobond repayment in 2020, accelerating its economic freefall. The money BDL borrowed seems to have ‘disappeared’—and with it the value of the private banks’ holdings—instead of being invested productively in Lebanon. Banking secrecy laws mean the whereabouts of missing billions remain unknown. How close Lebanon’s government is to bankruptcy is unclear as resistance to a forensic audit of BDL—upon which a requested International Monetary Fund (IMF) bailout of $11bn is conditioned—continues three years after the collapse, and after French and German authorities issued arrest warrants for BDL Governor Riad Salameh in May 2023.

For Lebanon’s population, the clock of displacement has started ticking; their lives have been disrupted in ways leaving them worse off in relation to a range of baselines in ways that produce displacement (Ali 2023). The economic collapse constrained people’s ability to generate income, and has stripped the banked half of the population of their assets. This is particularly important in Lebanon which has limited and underfunded public services. Healthcare, education, and electricity provision are largely privatized and expensive. Individuals rely on private resources to access good quality education in private schools and universities, on private hospitals for high standards of treatment, and on private generators to address national grid power cuts. Although private, these services rely on government support to function. Private hospitals rely on the government to pay the bills of public sector workers who have government health insurance, and the government regulates the price of medication in pharmacies. Private generators run on imported diesel fuel which was, until September 2021, government subsidized. In 2021, fuel shortages prompted altercations at petrol stations. While queues have abated, fuel remains extremely expensive. Power cuts are more frequent and last longer. A Lebanese youth who relocated to Cyprus explained that electricity provision was so unreliable that he feared he would be unable to receive calls to find work because of the difficulty of charging his phone (Chehayeb 2021).

## ‘Exodus’ from Lebanon?

Considering the severe impacts, mass emigration sounds feasible. In 2020, Lebanon’s Prime Minister and its most senior UN official both warned that Lebanese people could arrive on Europe’s shores, invoking the so-called ‘European refugee crisis’. The 2020 Arab Youth Survey found 77 per cent of Lebanese respondents wanting or planning to leave (ASDA’A BCW 2020). Meanwhile, media outlets echoed warnings from the Crisis Observatory at the American University in Beirut (COAUB) of Lebanon’s ‘third exodus’ (e.g. Dahan and Kanaan 2021; Taha 2021). The COAUB conducts valuable research about the effects of multiple crises in Lebanon. It warned in a press brief of protracted crisis and that Lebanon has entered a third wave of mass emigration, an exodus akin to that from C19th Mount Lebanon and the 1975–1990 civil war, evidenced by deteriorating conditions and accelerating professional emigration (COAUB 2021). The COAUB’s important work on deteriorating conditions in Lebanon, their likely protracted nature, and the emigration of professionals is not disputed. Rather, what is disputed is the assumption that the impacts of the current crisis are reproducing the mass emigration of those periods.

Beirut Today (Moussa 2022) reported a dramatic 446 per cent emigration increase, citing figures from Lebanese research centre Information International (INI 2022), itself another source of reports of an exodus. The increase of 79,000 in 2021 versus 17,700 in 2020 was widely circulated, but ignored pandemic travel restrictions in 2020, including the 3.5-month closure of Lebanon’s international airport. The figures from General Security, which manages Lebanon’s borders and issues passports, do not show for how long those people are away. Additionally, while the waiting list for new passports reached 150,000 (Damaj 2021), it is not evident who seeks emigration rather than travel. Travel durations aside, the financial crisis led the resource-poor Lebanese authorities to limit passport issuance to 40 per issuance centre per day (Damaj 2021), hindering possibilities for travel for those who do not already have passports.

Furthermore, included in warnings of an exodus are the intentions of people to travel expressed in surveys. Intentions, however, are not certain to produce emigration. A 2007 poll showed that 135,000 Lebanese youth had submitted some type of ‘emigration form’ (INI 2007), but annual emigration was under 19,000 in the following four years. In the next four years, 2011–2014, around 200,000 left (INI 2019) but this period saw violent spill overs from the Syrian conflict, and was not then termed an exodus, nor is it referenced in recent warnings. Additionally, intentions to emigrate can change. While 77 per cent of The Arab Youth Survey’s Lebanese respondents in 2020 said they were considering or actively trying to emigrate, only 48 per cent of their sample said the same in 2021 (ASDA’A BCW 2021), despite conditions in Lebanon deteriorating.^1^

Absent from the warnings are historical differences in financial liquidity. The COAUB warnings referred to mass emigration during the Lebanese civil war (1975–1990) and mass emigration in the late C19^th^. From 1860–1914, almost a third of Lebanon’s population emigrated, many to Latin America and the US (Issawi 1992: 31; Khater 2001: 70). But, says Khater (2001: 70), emigration happened at a time of stability and prosperity rather than destitution: people searched for ways to ‘guarantee, and possibly improve on, a standard of living they had grown accustomed to during the good times of the 1860s’. The population grew enormously from 1860 until WWI owing to peace and stability, unlike between 1830 and 1860 when famines, two major civil wars, and insurrections against Egyptian occupation took place (Khater 2001: 59–60). New legislation removed the power of political elites to restrict peoples’ freedom of movement (Khater 2001: 53), but emigration still required capital. Khater (2001: 55–56) details expenses, including travel and exit fees, and bribes demanded by Ottoman officials. In the 1880s and 1890s, peasants ‘mortgaged their land, borrowed from relatives, sold jewellery, got loans from richer neighbors. …’ demonstrating the availability of capital to people who were poor but not destitute (Khater 2001: 56).

Even during the mass emigration of the 1975–1990 civil war, capital was present for some years. Baumann (2019) calls 1975–1982 a period of resilience, in which time Lebanese migration to Arabian Gulf states led to rising remittances and industrial exports, creating a new diaspora bourgeoisie who had become Gulf contractors during the post-1973 oil boom. Despite government losses of port tariff income to militias, it still paid salaries, even establishing the Council for Development and Reconstruction during a lull in fighting (Baumann 2019: 63–64). Expectations of the war’s end in 1982 led to Lebanese-born Gulf contractors buying Lebanese banks, while Rafik Hariri began planning central Beirut’s reconstruction. But the war continued after 1982 and a period of collapse ensued. Coordinated currency speculation, the drying up of external militia funding, and the oil price collapse that diminished remittances were among its causes (Baumann 2019: 64). Labaki (1992: 607) estimates that 990,000 Lebanese emigrated during the civil war (although many returned at different times). A violent war economy, and changes to the Gulf states’ oil economies produced mass emigration (Labaki 1992) in circumstances very different to those of today’s capital scarcity.

## Migration: An Option for the Few

Only those with financial, social and cultural capital unaffected by the collapse have successfully left Lebanon. Others with scant resources, forced to attempt perilous boat journeys, have failed (Observer 2022). News stories feature professionals, like the exhausted Lebanese doctor awaiting her visa to relocate to the US because of unbearable pressures (AFP News Agency 2021). She can do so because her husband has US citizenship, demonstrating the importance of both the social capital of diaspora networks, and the cultural capital of her medical profession. Similarly, a doctor at Beirut’s St George hospital explained: ‘it’s a forced leave [sic], it’s not a decision I took because I wanted to leave, it’s a decision I took because the overall situation is bad.’ (France 24 2020) The reasons were: his lira salary, deteriorating conditions, and uncertainty. He moved from the US to Lebanon in 2018 with his family but will return because of those pressures (France 24 2020). The doctors’ syndicate told France 24 that 400 doctors left Lebanon in 2020. The chief nursing operations officer at the Clemenceau Medical Center in Beirut—a Johns Hopkins Medicine International affiliate—explained he conducted exit interviews for nurses almost daily: he said high calibre physicians and specialist nurses are leaving, and mostly moving to wealthy Gulf Arab countries, but some were headed for Europe or north America (Collard 2021). Training and experience from an affiliate of a globally prestigious medical institution constitutes cultural capital that enables these professionals to emigrate to evade the pressures of financial collapse.

At the American University of Beirut (AUB), 1500 faculty and staff left by June 2021 (Kenner 2021). Some were medical staff at AUB’s hospital, others used the cultural capital bestowed upon their CVs by AUB experience, and the prestigious degrees acquired to work there, to find work abroad. Additionally, academics often have social capital that facilitates their migration. Dr Charlotte Karam, a successful AUB scholar, moved to Canada—where she was born and has family ties—but will continue to work for AUB remotely (Perry 2020).

## Displacement: A Process that Begins in Place

Lebanon’s ongoing financial collapse has displaced the population in place. This may sound counter intuitive. However, the article moves beyond the conflation of displacement with the event of crossing a regional or international border, as is typical in media, policy, and most scholarship in RFMS. Displacement in place is also conflated with the eviction of tenants within regional boundaries (Sullivan 2017), something more accurately considered as forced relocations (Ali 2023). Instead, the article frames displacement as a disruptive process beginning in place, building upon scholarship that engages with it as such, some from outside of RFMS (Feldman *et al*. 2004; Marfleet 2011; Mollett 2014; Celestina 2016; Safransky 2017; Vaz-Jones 2018; Ali 2020, 2023). Forced mobility’s counterpart is forced immobility (Lubkemann 2008) or locational captivity (Penz 2006), similarly potential outcomes that should not be conflated with the preceding causative process.

Displacement is misleading semantically, suggesting being physically dislodged from a geographical location. This relates to an event-centred perspective where displacement starts with movement from one place (home) to another (site of refuge) which dominates thinking on displacement while obscuring precursive and processual complexities (Marfleet 2011: 281). In conflict scenarios of prolonged generalized insecurity, more readily associated with forced migration, a complex process typically precedes decisions to leave (Marfleet 2011: 281). In Iraq, people enacted strategies of avoidance, changing routines to avoid dangerous places, over months or even years often rejecting the option of leaving their homes until pressures became too intense, forcing them to leave (Marfleet 2011: 281). Such processes can be overlooked by focusing primarily on moments of departure, and subsequent migration trajectories. Importantly, similar pressures are experienced by people who remain in place in stratified, gendered, and generational ways (Lubkemann 2008). They are displaced—subject to coercive pressures through which a more powerful agent or group leaves them worse off in relation to key baselines (Ali 2023)—but not visible in RFMS because they have not crossed a border, or travelled far enough to become ‘internally displaced persons’. Displacement begins in place, and it can force people to relocate as well as immobilize them. It can produce the outcome of involuntary immobility, potentially leaving people in settings of diminished capacity for action, particularly difficult for those reliant on mobility (Lubkemann 2008). For most of Lebanon’s population, the capacity to emigrate has been diminished. But this is an *outcome* of a displacement process which has stripped them of their assets and prospects.

Moving beyond conflating displacement with its outcomes, recent scholarship acknowledges its processual in situ nature. Celestina (2016: 368) argues that the ‘clock’ of displacement starts before the departure. While departure is commonly understood as when displacement begins, this understanding helps to conceal state complicity in ‘complex political, social, and economic contexts that have generated displacement processes’. This is highly pertinent in Lebanon where political elites are complicit in the causes of financial collapse and the exacerbation of its consequences. Vaz-Jones (2018: 714) shows how displacement is not a ‘single moment’ but an ongoing process, destabilizing people’s lives and obstructing their access to land and resources. Vaz-Jones (2018) alerts us to scholarship reframing displacement as a process happening in place, not involving physical relocation, but rather of displaced futures resulting from constraints on livelihoods and cultural practices (Mollett 2014: 30). People experience displacement without moving (Nixon 2011: 19) by losing the land and resources beneath them. Meanwhile, Safransky (2017: 1089) says displacement means loss of public services and resources, of ‘being left behind rather than forcibly moved’. Additionally, it can mean ‘relations of exclusion that set new boundaries for people’s physical and social movement’, with examples including the loss of ‘welfare benefits and other entitlements, suffering discrimination or ostracism, or having one’s civil rights or property agreements suspended’ (Feldman *et al*. 2004: 9). More recent work identifies the constitutive components of displacement, conceptualizing it as coercive, processual, and disruptive to valued ways of living and functioning (Ali 2023). Coercive agents, institutions, or other organizations, leverage power imbalances in morally unacceptable ways (Ali 2023). People are left worse off in various ways including access to economic resources, to infrastructure provision, healthcare, or other resources (Ali 2023).

In Lebanon, conflating displacement with forced migration would mean focusing on the small minority who have been able to leave, rendering invisible the myriad ways in which people have been displaced without moving, such as by having access to their savings denied, forced to watch them become worthless with severe consequences for their present and future circumstances. Meanwhile, public service deterioration has accelerated, along with people’s purchasing power. BDL, Lebanon’s central bank, with its role in causing and exacerbating the collapse, is a key coercive agent in this displacement, using its power to force the population to endure the burden of its financial misconduct. Coercion, not only in terms of threats in violent conflict scenarios, is a definitive component of displacement (Ali 2023). Coercers create or leverage power imbalances to foreclose possibilities for action of the coercee, and possibly in ways that the coerced cannot resist (Anderson 2010 in Ali 2023). The coercer may be an institution that uses a power imbalance to determine what an agent will or will not do, disrupting their ability to act (Anderson 2010). In the displacement of Lebanon’s population BDL has leveraged its powers to prevent depositors from exercising their will to choose what to do with decades of savings in a time of crisis. The BDL has resisted deep audits of its practices that could have led to assistance and action that may have lessened the burden, and the displacement pressures, upon the population.

## Displacement in Place

I provide an overview of certain consequences of the collapse that comprise displacement in place. Had a million Lebanese citizens already arrived in Europe to escape these consequences, they might be the focus of attention in RFMS because of the movement-centred understanding of displacement. By considering recent scholarship on displacement, it is possible to see how financial collapse has displaced Lebanon’s population in place, because it is not a ‘single moment’, but an ongoing process of lives being destabilized and access to resources lost (Vaz-Jones 2018: 714). People are experiencing displacement without moving (Nixon 2011: 19) having had their assets stolen during alarmingly high and widespread inflation (CAS 2023), and consequentially being ‘left behind rather than forcibly moved’ (Safransky 2017). Power imbalances have been coercively deployed by private banks, the central bank, and a deliberately inert political elite, to rob Lebanon’s population of their assets and their public services, disrupting their lives against their will (Ali 2023). The notion of baselines is also organizationally useful, and the following shows how economic baselines have an array of further implications. Not only has this displaced the population in the present, but it has also created what Mollett (2014) calls displaced futures.

## Economic Baselines

Lebanon’s GDP was USD$55bn in 2018 but USD$23bn in 2021, and per capita income fell from $9000 to $4000 in the same period (World Bank 2023). But this is only a partial indicator as an average. Inequality is very high in Lebanon. The Beirut-based United Nations Economic and Social Commission for Western Asia (UNESCWA) reports that households experiencing multidimensional poverty, i.e. across more than one dimension, increased from 42 per cent in 2019 to 82 per cent in 2021. This index includes six dimensions—education, health, public utilities, housing, assets and property, as well as employment and income—with 20 indicators across the dimensions (UNESCWA 2021: 3). The UNESCWA estimated a 55 per cent headcount poverty rate (the poor and extreme poor) in May 2020, up from 28 per cent in 2019. The lower middle-income bracket shrank from 45 to 35 per cent. The affluent and upper middle-income brackets shrank from 26 per cent to under 10 per cent of the population (UNESCWA 2021), while COAUB estimates that 80 per cent of the population are food insecure (BBC 2021). The currency’s collapse has seen the cost of imported goods rise immensely, but even locally grown food is more expensive as costs of imported fuel, fertilizers, and pesticides upon which farmers rely has risen, accelerated by the end of subsidies. Official statistics point to this worsening with inflation of 269 per cent on consumer goods from April 2022 to April 2023 (CAS 2023).

## Education

It is in education that displacement in place can be seen not only as increasing constraint on present livelihoods, but also as displaced futures (Mollett 2014). Among them are what Lubkemann (2008: 470) identified as highly consequential ‘key social life projects’, crucial to the social well-being of individuals. Their disruption can sabotage future social life projects. Disruption to high school education jeopardizes a university education, and thus entry into the professional class and access to associated cultural and social capitals. Disruption to education can thus also produce relations of exclusion that set boundaries for people’s social movement in ways constitutive of in situ displacement (Feldman *et al*. 2004). Not only has the poverty rate risen for students—at university, the multidimensional poverty rate is 63 per cent, and 87 per cent among students with the lowest levels of educational attainment—but the crisis has limited access to education overall. Students have suspended or abandoned studies, or switched to institutions that are either free or less expensive, but of comparably lower quality. Lebanon’s education sector is highly privatized. Underinvestment in public education sees parents with the means to do so typically educating their children privately in stratified schools of different cost and quality. Parents were already struggling to pay fees in 2019, such as in rural areas furthest away from Greater Beirut where up to 25 per cent of parents were unable to pay fees (Rahhal 2019). Financial collapse has exacerbated this, leaving more families unable to pay.

In higher education students were already struggling to pay rising fees before 2019 with 62 per cent enrolled in fee-paying universities. The Lebanese University is the only free and public university, and chronically underfunded, despite hosting 38 per cent of Lebanon’s students in 2017. When the collapse began, private universities allowed students to pay fees in instalments, and in local currency, but in 2022 tuition fees were dollarized (universities describe it as a survival strategy) putting access to higher education in further peril (Sabaghi 2022). The AUB now accepts tuition fees only in dollars. Few will be able to afford studying there with undergraduate tuition ranging from around $11,000 per year in the arts to $40,000 per year in medicine (AUB 2022). At the relatively less expensive Saint Joseph University, European accredited degrees cost from US$35,000 in social sciences to $140,000 in medicine. While some might welcome the economic relief from a shift to the Lebanese University, others will lament the lack of international recognition that they could expect with a degree certified by US or European authorities. Losing this for many is a form of displacement—displaced futures (Mollett 2014) and disruptions to key social life projects (Lubkemann 2008) which they may have previously expected to lead to further study and professional development internationally.

## Healthcare

Displacement in place can be about being left behind rather than being forced to move, and losing access to services (Safransky 2017), evident with diminishing access to healthcare in Lebanon. The share of households deprived in healthcare rose from 9 to 33 per cent in 2021, and this includes access to medicines, medical services and insurance, with 52 per cent of the population in 2021 unable to obtain medicines, according to UNESCWA (2021). It is not only that people cannot afford to pay for healthcare as a result of a severe economic decline, it is that the nature of the crisis means that the Lebanese government, the largest provider of health insurance, is almost bankrupt, thanks to the misconduct of BDL. Lebanon’s healthcare largely consists of insurance programmes for government employees to access subsidized private healthcare (Lebanon Support 2016). The government is heavily indebted to private hospitals (representing 82 per cent of national capacity) and public hospitals received no government payments in 2019 (HRW 2019), including the main CV19 treatment hospital, Rafiq Hariri University Hospital (RHUH) whose staff enacted strikes in 2020 against poor working conditions and non-payment. The government accumulated $1.5.bn of debt to private healthcare institutions by 2019 (BN 2020), and the Syndicate of Private Hospitals’ head said 20 from 126 hospitals may consequently close (Akoum 2020). The Association of Private Hospitals in Lebanon decided in July 2020 to allow members to suspend non-emergency and non-urgent admissions without pre-payment, with important exceptions (kidney dialysis, other life-threatening cases) (Brito 2020). Healthcare deteriorated further in 2021. Fuel shortages forced some hospitals to run at 50 per cent capacity, cancelling operations for fear of generator outages (WHO 2021). In August 2021, a judge ordered thousands of litres of fuel, confiscated from smugglers, to be sold with subsidy to major hospitals in Beirut after two issued distress calls; but authorities failed to comply (Amnesty International 2021). The Director General of the World Health Organisation (WHO) and its Regional Director for the Eastern Mediterranean issued a joint statement after visiting Lebanon in September 2021, expressing alarm at supply shortages, and brain drain of medical professionals, with 40 per cent of doctors and 30 per cent of nurses estimated to have left the country for work abroad by 2021 (WHO 2021). The departure of experienced medical professionals is also likely to have negative long-term consequences for healthcare provision and the population more broadly.

## Resistance to Displacement: Initiatives by Lebanese and Refugee Populations

Like other processes, displacement involves phases and counter phases, neither evenly balanced nor neatly sequenced. Counter phases may consist of strategies of evasion, coping, and resistance to the threats and pressures intended to constrain or exclude people (Ali 2023: 1099). Resistance and threat evasion are, however, ‘… contingent upon resources and power relations which relate to social identities and class.’ (Ali 2023: 1099). Discussing them in the detail they deserve is beyond the article’s scope, but I summarize some in their different forms.

Foremost were nationwide cross-sectarian protests in response to proposals for new taxes to burden the population with the cost of government mismanagement and corruption. The most provocative of these was the ‘WhatsApp’ tax, a $0.20 daily charge to use a free communications app. The backlash saw the government quickly backtrack but nationwide protests began against elite corruption and broader systemic failures. The 17 October 2019 Revolution saw protestors occupying public spaces, demanding meaningful reform. Protests crossed social lines, with angry discontented middle and working classes coming together against corrupt elites (Khattab 2022). However, the pandemic gave the government a pretext to ban gatherings and protests for some time, and to destroy Beirut’s protest camps at Martyrs’ Square. Elites stubbornly refused to enact meaningful reforms. Despite the government requesting an $11bn bailout from the IMF in 2020, which in turn conditioned it on forensic auditing of the central bank and economic reforms, the government and BDL continue to resist forensic auditing. Although a preliminary deal with the IMF was reached for $3bn of assistance in exchange for economic reforms, the reforms remain pending (L’Orient 2022). In 2020, Lebanese civil society groups, former officials, and analysts petitioned the IMF not to provide support without political as well as economic reforms (Sewell 2020) but it appears not to have listened.

Protests continue across the country, albeit not on the scale of 2019. At times banks were targeted as people vented their anger at buildings. In 2022, several depositors took matters into their own hands, by making armed withdrawals (often wrongly reported as ‘robberies’), demanding access to their own dollars at gunpoint (Stephan 2022). While such incidents are increasing, including individuals withdrawing money to pay for their relatives’ medical bills, (MEMO 2022) little has changed nationally in terms of depositors’ restitution.

In 2022 independents won 13 from 128 seats in parliamentary elections, with the Hizballah-led bloc losing its majority. There is not space for full discussion here, but a disparate group of independent candidates defeated status quo politicians in 13 seats (TLPP 2022). However, new independents are not a cohesive bloc, including reactionary and progressive candidates, and progressive change is not guaranteed (TLPP 2022). In October 2022, President Aoun’s term ended with no agreed successor, making protracted political paralysis likely, such as when parliament could not agree upon a President between May 2014 and October 2016. Since 2022, parliament has failed 12 times to agree on Aoun’s successor.

## Community and Refugee Initiatives

Considering displacement as a process that happens in place, rather than conflating it with forced movement, can also help to frame the effects of the collapse on refugees in Lebanon. It has not forced hundreds of thousands of Palestinian refugees in Lebanon to leave, in the way that Palestinians were forced out of Libya in 2011 (Fiddian-Qasmiyeh 2012) and Iraq (Ali 2020), but their lives have been coercively disrupted in ways that displace them (Ali 2023). It is a process which does not necessarily stop for refugees after they cross borders, and Fiddian-Qasmiyeh (2020) refers to ‘overlapping precarities’. Generations of Palestinians forced to flee to Lebanon, and subject to labour market, and other forms of discrimination from Lebanese authorities, now face additional pressures from financial collapse that exacerbate their already precarious lives (Fiddian-Qasmiyeh 2020), as do Syrians. Forced from their homes in Syria by violent repression and conflict since the 2011 uprising (Ali 2015), in Lebanon they have been subject to increasing restrictions since 2014 (Betts *et al*. 2021) and harassment in recent years that has included escalating hate speech, surveillance, raids and arrests, as well as detention and abuse (SAWA 2023).

Lebanese healthcare systems excluded Palestinian and Syrian refugees long before financial collapse, and the structure of international aid divides refugee populations into those who can seek aid from UNRWA, the agency for Palestinian needs, and UNHCR, for all other refugees (Fiddian-Qasmiyeh 2020). Both agencies’ programmes are underfunded in Lebanon. Medical Aid for Palestinians notes before the crisis 65 per cent of Palestinians in Lebanon lived below the poverty line, compared with 28 per cent of the Lebanese population, making Palestinians more vulnerable to economic shocks (MAP 2021). Community-led initiatives have been among those to help refugee families cope. In north Lebanon’s Beddawi Camp, residents include Palestinian, Iraqi, Kurdish and Syrian refugees, as well as Lebanese. The pandemic prompted community-led efforts to share information and resources to protect residents from covid and hunger (Fiddian-Qasmiyeh 2020: 31). Residents of Mar Elias and Burj al-Barajneh camps nearby, and US-based Palestinian organizations, joined the initiative. While successful in their relief goals, they could not withstand the economic impacts of covid layered onto pressures of financial collapse (Fiddian-Qasmiyeh 2020).

Beyond Beddawi, and around Lebanon more widely, are initiatives of joint-Lebanese-Syrian community-focused civil society organizations such as Basmeh & Zeitooneh—(B&Z) and SAWA, both focused on relief and development support for Syrian refugees as well as vulnerable Lebanese groups. SAWA, which means ‘together’ in Arabic, was founded in 2011 and B&Z, meaning the ‘smile and the olive’, in 2012 after Syrian refugees arrived. Both have witnessed financial collapse exacerbate existing vulnerabilities. On a visit to Lebanon in 2022, staff and beneficiaries—Lebanese and Syrian—explained some of the deepening issues. Close to a SAWA office in the Beqaa valley, residents of an informal camp (official Syrian camps are not permitted) for Syrian refugees were facing eviction as the landowner raised the rent for the land and demanded payment in US dollars. A bakery that SAWA ran, a source of employment and bread, was not operating at the time owing to pressures associated with financial collapse, including the end of government subsidies for fuel and flour for bread. But initiatives to support refugee populations continued, such as the clothing workshop that employed Syrian women in the Beqaa, generating income through the sale of their products in Beirut.

The multiple precarities have been exacerbated by the misconduct of BDL, further displacing already vulnerable populations in place. The UNESCWA (2021) noted 77 per cent of Lebanese lived in poverty, while UNHCR reported 90 per cent of Syrians lived in poverty (UNHCR VASyR 2021). Refugee populations, already struggling, are now even worse off than they were before the collapse in terms of their abilities to access and afford food, medication, and other essential goods.

## Conclusion

A lack of capital has taken away the capacity of most people in Lebanon to emigrate, regardless of their intentions. A specific feature of the financial collapse, the absence of widely accessible capital liquidity, is one way in which the current period differs from earlier historic periods of emigration. But displacement in place is not about wishing to move and not being able to. Immobility can be one of the outcomes of displacement. Displacement is a process which disrupts the ability of people to live in valued ways by a coercive party that creates a power imbalance or leverages an existing one. It begins before people move, or try to move but fail, and it is a process that deserves greater attention in RFMS than it currently receives. Had many thousands of people seeking refuge from deprivation in Lebanon already arrived in Europe, Lebanon’s collapse might have already received scholarly attention. Without a recapitalization of the economy, the continuation of the current situation of migration being limited to very few is likely. The recent US-sponsored gas agreement between Lebanon and Israel could lead to future recapitalization, as could the effects of the recent Iranian-Saudi rapprochement. But the kleptocratic record of Lebanese politicians and the corrupt self-interested practices of the central bank offer little hope that wealth will be redistributed in ways considerate of societal needs. Regardless of future emigration numbers, the major transformations underway in Lebanon warrant attention from our field as they constitute the displacement in place of a national society, and further displacement of existing refugee populations. In the parlance of scholars already engaged with the processual nature of displacement in place, the clock of displacement in Lebanon is already ticking (Celestina 2016), lives have been destabilized (Vaz-Jones 2018), futures displaced (Mollett 2014) and relations of exclusion have set further boundaries for people’s physical and social movement (Feldman *et al*. 2004). People are being left behind rather than forced to move (Safransky 2017). Political elites in the government and banking sector have forced upon the population a set of circumstances that prevent the majority of the population from living and functioning in ways that they value and in doing so have displaced them in place (Ali 2023). Beyond Lebanon, scholars of RFMS ought to consider the displacement processes that precede the forced migration upon which most scholarship in the field remains centred.

## Data Availability

Due to issues of confidentiality the data underlying this research cannot be made publicly available.

## Footnotes

1 The 2022 edition of the Arab Youth Survey does not provide a country-level breakdown of survey results but states that 53% of youth surveyed in the Levant (which means Iraq, Jordan, Lebanon, Palestine, Syria and, unusually, Yemen) are actively trying to or considering emigrating to another country (ASDA’A BCW, 2022).
